# Inter-Rater and Intra-Rater Reliability of the INSPECT (Interactive Nutrition Specific Physical Exam Competency Tool) Measured in Multi-Site Acute Care Settings

**DOI:** 10.3390/healthcare10020212

**Published:** 2022-01-21

**Authors:** Sunitha Zechariah, Jennifer L. Waller, Judith Stallings, Ashley J. Gess, Leigh Lehman

**Affiliations:** 1 College of Allied Health Sciences, Augusta University, Augusta, GA 30912, USA; jstallin@augusta.edu; 2 Medical College of Georgia, Augusta University, Augusta, GA 30912, USA; jwaller@augusta.edu; 3 College of Education, Augusta University, Augusta, GA 30912, USA; agess@augusta.edu; 4 School of Occupational Therapy, Brenau University, Gainesville, GA 30501, USA; llehman@brenau.edu

**Keywords:** competency, inter-rater reliability, intra-rater reliability, internal consistency, nutrition-focused physical exam, registered dietitian nutritionists

## Abstract

Even as new medical modalities, diagnostics, and technologies are rapidly changing healthcare, providing patients with safe, high-quality care remains the central focus. To provide safe patient care, healthcare providers are obligated to demonstrate and maintain the necessary competence. As more healthcare disciplines move toward a competency-based education model, it is essential to extend the competence verification from the academic educational level to the patient’s bedside. The nutrition-focused physical exam (NFPE) is a competency recently adopted by registered dietitian nutritionists (RDNs) for assessing patients’ nutritional status. Being a newly acquired skill, validated tools are required to measure NFPE competence during routine clinical practice. The Interactive Nutrition Specific Physical Exam Competency Tool (INSPECT) is a new tool developed specifically to observe and measure RDNs’ NFPE competence in clinical settings. The INSPECT was designed and validated for content using expert RDNs’ input in the first and second phases of the study. This current study aimed to assess the reliability of the INSPECT through multi-site observations by clinical supervisors evaluating RDNs’ NFPE competency during patient assessment. The INSPECT exhibited good inter-rater reliability (ICC = 0.78 for the first assessment and ICC = 0.68 for the second assessment), moderate to strong intra-rater reliability for 37 of 41 items (Spearman rho = 0.54 to 1.0), and excellent internal consistency (Cronbach’s α = 0.86 for the first assessment and α = 0.92 for the second assessment). In total, 10 out of the 11 INSPECT subsets showed good to excellent internal consistency (α ranging from 0.70 to 0.98). The results demonstrate that the INSPECT is a reliable tool, is stable over time, and has good agreement and excellent consistency between raters. The INSPECT can be a valuable tool to measure, promote and maintain RDNs’ NFPE competence in authentic acute care settings.

## 1. Introduction

The assessment of healthcare providers’ clinical performance during their everyday practice is crucial for the delivery of reliable, safe, and high-quality patient-centered care [1,2,3]. Progressively increasing patient-care responsibilities and advances in medicine require providers to be astute and competent in their clinical skills [1,4]. Healthcare professionals acquire education and competence through required academic programs and then are qualified through an examination that tests their knowledge and application skills. Once qualified to practice after passing the credentialing exam, some healthcare professionals such as RDNs, physical therapists, occupational therapists, and nurses can provide patient care for the remainder of their career with the sole requirement of maintaining their competency through the required continuing education hours within their area of expertise [5,6,7,8]. This system presumes that these healthcare providers remain competent by staying abreast with new knowledge and science relevant to their practice [9]. The presumption of continued competence of healthcare professionals without verification, particularly at the bedside is imprudent in healthcare service as it may compromise optimal patient care [4,9]. Regulatory agencies and accrediting organizations that certify hospitals on quality standards expect hospitals to conduct annual competence assessments on all staff to ensure that the staff is proficient to perform patient responsibilities [10]. In addition, many healthcare professions including dietetics are moving toward adopting a competency-based education model, requiring the need to develop and disseminate competence assessment tools that reflect authentic everyday clinical practice [1,4,11]. Ongoing competence verification is thus critical for all healthcare providers including allied health professionals.

Registered dietitian nutritionists (RDNs) are allied health professionals who support the interdisciplinary patient care team by assessing the patients for malnutrition and other nutrition-related deficiencies. The role of RDNs has been evolving as the scope of practice for RDNs has expanded to include advanced skills to take on larger responsibilities within the interdisciplinary team [12,13]. One such skill that RDNs have recently incorporated in their clinical practice is the nutrition-focused physical exam (NFPE). RDNs employ NFPE to accurately identify protein-calorie malnutrition, nutrient deficiencies, and other nutrition-related concerns and utilize the information to appropriately assess and treat patients [14,15,16,17,18,19]. As RDNs adopt new skills such as NFPE in their clinical practice, assessing their competence to verify their level of performance becomes imperative.

Measuring knowledge through written or computer-assisted tests alone do not provide the full picture of RDNs’ NFPE competency during a patient’s nutrition assessment. A comprehensive competency assessment must include direct observation of hands-on performance along with other competencies such as patient interview skills, communication skills, safe hand hygiene practices, and so on [20,21,22]. Appropriate, reliability tested and validated competency tools that accommodate direct observation in a workplace environment are necessary to verify the initial and ongoing competence of RDNs including the NFPE performance [21,22,23,24]. Although the need for a validated competency tool to measure RDNs’ regular NFPE performance is clearly evident, such tools are severely limited with only one such tool recently made available in the form of a competency checklist [24]. As far as the authors know, at present, there are no reliable, valid NFPE competency tools that are developed based on direct input from expert RDNs practicing in the field and are designed to provide interactive scoring. Therefore, the need arose to systematically develop and design an interactive tool, the INSPECT (Interactive Nutrition Specific Physical Exam Competency Tool), and scientifically test it for reliability and validity measures to evaluate RDNs’ NFPE competence.

The initial NFPE components required to construct the INSPECT were acquired through expert focus group discussions using which a preliminary version was developed. This preliminary version of the INSPECT was then tested for content and face validity utilizing the Delphi methodology. An outline of these phases of the study is described in the methods section. Having established acceptable levels of the face and content validity for the INSPECT, the next, logical step in tool development was to examine the reliability and other appropriate measures of validity. Therefore, the third phase of this study aimed at measuring the inter-rater and intra-rater reliability and internal consistency of the final version of the INSPECT through field tests at multi-site, real-life acute care settings among practicing RDNs.

## 2. Methods

### 2.1. Initial Phase of the INSPECT

The INSPECT was developed in the first phase of the study with 70 items identified by seven content and practice experts who explored the NFPE components through technology-based focus group discussions. The methodology of item generation and identification of NFPE components is described in detail in a previous publication [25]. Using the items generated in the first phase of the study, a preliminary version of the INSPECT that incorporated all areas of physical assessment was developed. In the preliminary version, the tool items were categorized into 13 subsets based on a head-to-toe sequence. The 13 subsets were (1) preparation and initial steps, (2) head and hair exam, (3) face exam, (4) eye exam, (5) mouth and oral cavity exam, (6) neck exam, (7) clavicular/thoracic region exam, (8) abdominal exam, (9) back/scapular region exam, (10) upper extremities exam, (11) lower extremities exam (12) functional grip strength exam, and (13) bedside manner and etiquette. Each subset consisted of a varying number of items ranging from 3 to 13 depending on the exam area. Each NFPE item under each subset was provided with performance indicators along with a scoring scale. The preliminary version of the INSPECT was designed using Microsoft Excel^TM^ (2016) with formulas embedded to calculate scores automatically. The INSPECT is set up to compute scores for each subset, the overall NFPE score, the overall percentage, the overall total points possible, and the overall total items missed.

### 2.2. Second Phase of the INSPECT

The second phase of the study focused on gauging face and content validation of the preliminary version of the INSPECT using the Delphi methodology. Seventeen experts participated in two rounds of Delphi and rated the INSPECT independently and anonymously for content and face validity. An 8-item dichotomous scale with ‘clear’ and ‘not clear’ was developed by the authors to measure the overall appearance of the INSPECT. The content validity was rated using a 5-point Likert scale (1 = not important, 2 = sometimes important, 3 = important, 4 = very important, and 5 = essential). In addition to the face and content validity rating scales, experts were invited to provide suggestions on any aspect of the INSPECT to enhance the design, the content, and the scoring aspects of the tool. The expert consensus or internal consistency of the expert group for face validity was found to be acceptable. Content validity for the INSPECT showed excellent internal consistency and inter-rater agreement in each of the Delphi rounds. Based on the expert consensus and open feedback from the experts, a total of 41 items and 11 subsets were identified and included in the final version of the INSPECT. A detailed account of the second phase of the Delphi consensus study with evidence of face and content validation is given in a previous publication [26].

### 2.3. Third Phase of the INSPECT

This third phase of the study utilized the final version of the INSPECT, which included 41 items and 11 subsets (Table 1). The subsets are (1) preparation and initial steps (5 items), (2) head and hair exam (2 items), (3) face exam (3 items), (4) eye exam (3 items), (5) mouth/oral cavity exam (5 items), (6) clavicular/thoracic region exam (6 items), (7) back/scapular region exam (2 items), (8) upper extremities exam (5 items) (9) lower extremities exam (4 items) (10) functional grip strength exam (2 items), and (11) bedside manner and etiquette (4 items). Each of the items in the tool has a performance indicator that described how the exam should be performed on a patient. This performance indicator acted as a guide to the rater during direct observation of the RDNs performing NFPE.

The scoring structure for the final version comprised of a 4-point scale of ‘complete = 2’, ‘partially complete =1’, ‘incomplete = 0’ and ‘not applicable = NA’. The raters rated the NFPE performance as ‘complete = 2’ for performing a specific item accurately, ‘partially complete =1’ for performing an item partially accurate, ‘incomplete = 0’ if the specific item was not performed or was performed inaccurately, or NA if that item did not apply to the patient. The tool was set to calculate scores for each subset based on whether an item is rated as ‘complete’ or as ‘partially complete or as ‘incomplete’. The INSPECT calculated subset scores by adding all the items that score ‘complete = 2’ and ‘partially complete =1’ for that subset. Items that were not applicable (‘NA’) were not included in the calculation of scores. Each of the 11 subset scores was summed to provide the overall INSPECT score. The tool also calculated the overall total points possible, and the overall total items missed. Using the overall INSPECT score and the overall points possible, an overall percentage is computed.

### 2.4. Study Population for the Third Phase

This study recruited clinical nutrition supervisors (raters) to observe RDNs (performers) performing NFPE on patients and rate the performance using the INSPECT. To standardize the rating process, only clinical supervisors and RDN performers who met the inclusion criteria were selected to participate in the study. Clinical supervisors with a minimum of 2 years of clinical management experience, 2 years of clinical dietetic experience, and one year of NFPE experience in an acute care setting were recruited as raters. Clinical RDNs with a minimum of 1 year of experience in clinical practice and a minimum of one year of experience performing NFPE in an acute care setting were recruited as performers in the study. All RDNs and clinical supervisors were required to meet all inclusion criteria for clinical practice and NFPE experience to be included. Assessments were only completed by clinical supervisors as they typically evaluate the competency of RDNs in real-life clinical settings, which was also affirmed during the expert focus group discussions in the first phase of the study. The desire to mimic real-life workplace competency assessments and the need to minimize variability in assessments precluded the use of RDN peer-to-peer evaluations.

### 2.5. Study Design and Clinical Setting for the Third Phase

A priori sample size calculation was completed to estimate a Cronbach’s alpha of 0.8 at a significance level of *p* < 0.05 and power of 0.80 using SAS 9.4 [27,28]. A minimum of 40 RDN assessments were required to test if Cronbach’s alpha is different from 0.50 at a significance level of *p* < 0.05. Raters (clinical supervisors) were recruited from acute care hospitals around the United States during March 2021. Raters were fully informed of the purpose and procedures of the study and only raters who provided written consent were included for participation in the study (April to July 2021). This study was approved by Augusta University institutional review board (1721423-2) and was deemed as exempt from full board review.

For data collection purposes, the INSPECT was embedded within the Qualtrics^XM^ platform (Qualtrics, Provo, UT, USA). Raters and performers were assigned identification codes for tracking purposes. Raters were provided detailed instructions on the functionality and the rating scale of the INSPECT and how to perform the ratings via email and through Qualtrics. For each observation, raters were asked to indicate whether an item was fully performed, partially performed, or not performed on the INSPECT using the performance indicators as a guide. When an RDN performs a certain item on the tool, the raters were instructed to assign a score of ‘2’ to indicate completion of the item. When an RDN fails to perform a certain item or uses the wrong technique, the raters were asked to assign a score of ‘0’ to denote that the item was not completed. When the RDN partially completes the item, the raters were instructed to give a score of ‘1’ to indicate that the item was only partially completed. For any item that did not apply to the patient or was unable to be performed on the patient, raters were asked to assign ‘not applicable’ or ‘NA’ for that item. In addition to the first assessment, raters were asked to repeat their observation on the same participants after 2 weeks to measure intra-rater reliability. A 2-week timeframe between assessments was considered appropriate as it was long enough to avoid performers from remembering the previous observation and rating [29].

### 2.6. Statistical Analysis for the Third Phase

#### 2.6.1. Inter-Rater Reliability

Inter-rater reliability for the INSPECT is the extent to which different raters provide a consistent estimate of measurement on RDNs performing NFPE on patients in an acute care setting [30]. To determine the inter-rater reliability of the INSPECT within first or second assessments, a one-way random-effects intraclass correlation coefficient (ICC) model was selected with 95% confidence intervals. The ICC model was the preferred statistical measure of reliability since it reflects both the degree of correlation and the agreement between rater measurements. As this is a multi-center study, one rater assessed a subgroup of RDN performers in one hospital while another rater with similar characteristics assessed a subgroup of RDN performers in another hospital and so on. Since the physical distance between hospitals prohibited the use of the same set of raters to rate all RDN performers, a one-way random ICC model where people effects are random was utilized [30,31]. The ICC values between 0.5 and 0.75 was set to indicate moderate reliability, values between 0.75 and 0.90 indicate good reliability, and values greater than 0.90 was set to denote excellent reliability based on Koo and Li’s interpretations [31]. A significance level of 0.05 (*p* < 0.05) was used to determine statistical significance.

#### 2.6.2. Intra-Rater Reliability

Intra-rater reliability or test-retest reliability for the INSPECT is the measure of the consistency of a rater at time 1 (first assessment) and at time 2 (second assessment in two weeks). To assess intra-rater reliability of the INSPECT, raters were asked to rate the RDN performing NFPE on a patient for the first time and then rate the same RDN performing NFPE two weeks later. Intra-rater reliability was analyzed for each item using Spearman rho correlation for non-parametric data and a test-retest correlation of 0.80 or greater was considered indicative of good reliability [30]. A Wilcoxon Signed-Rank test was also performed on each item to determine if statistical differences existed between the first and second assessments. A non-significant Wilcoxon Signed-Rank test was set to indicate good intra-rater reliability for each item.

#### 2.6.3. Internal Consistency

Internal consistency for the INSPECT is the correlation between multiple items that are intended to measure the same NFPE construct. Cronbach’s α is a common measure of internal consistency and is appropriate for tools with Likert Scale rating such as the INSPECT. Hence, Cronbach’s α reliability coefficient was used to measure internal consistency. An overall Cronbach’s alpha was calculated for the first assessment, for the second assessment, and for each subset of the INSPECT. A minimum of Cronbach’s α of 0.70 was considered as acceptable consistency and an α of 0.80 as good and >0.9 was considered as highly consistent [30,32].

### 2.7. Demographic Data for the Third Phase

Demographic data of age, gender, race, highest degree attained, job role, years of clinical practice, years of NFPE practice, practice location were obtained from RDN performers and raters. In addition, the number of years of clinical nutrition management and number of RDNs supervised was obtained from the rating supervisors. Descriptive characteristics of study participants including medians and interquartile ranges, frequencies, and percentages were calculated to determine if the data are reflective of the desired population [33]. The reliability and demographic data were analyzed using SPSS Statistics software, version 27 (IBM SPSS, Inc., Armonk, NY, USA).

## 3. Results

A total of 31 clinical supervisors (clinical nutrition managers and lead RDNs) responded to the invite to be raters of the INSPECT. Of the 31 respondents, six of them did not complete all the required details and did not provide consent, one person gave consent twice and hence was a duplicate, one rater withdrew, and one rater did not meet the inclusion criteria. As a result, a total of 22 raters were recruited for the study, of which eight raters failed to provide assessments during the study period. In the end, 14 raters from 14 different acute care hospitals representing diverse geographic locations provided RDN NFPE assessments for the INSPECT, resulting in a 64% response rate. A total of 57 first assessments were received from the raters (*n* = 14), however, one rating had several missing data and was eliminated from the study, resulting in 56 first assessments. Three raters (*n* = 3) repeated the observation at 2 weeks on the same RDN performers and provided 16 second assessments. In total, 72 RDN assessments (56 first assessments and 16 second assessments) were received, thus meeting the sample size requirement established a priori.

Demographic characteristics of the raters presented in Table 2 showed a median age of 35 years (range = 28.7–52.5), a median clinical dietetic experience of 11.5 years (range = 5.8–17), a median NFPE practice experience of 5 years (range = 3.8–5.3), a median clinical supervision experience of 6 years (range = 4–10), and a median number RDNs supervised as 5.5 (range = 4–11). A majority of participants were female (86%) and were of White, non-Hispanic ethnicity (100%). This study population is representative since the profession of dietetics has been dominated by females (93%) of White, non-Hispanic ethnicity (82%) for several decades [34]. Most had completed a graduate degree (64%), were employed as regional clinical or clinical nutrition managers (78.6%), and worked in the inpatient setting of acute care hospitals (100%).

### 3.1. Reliability of the INSPECT

#### 3.1.1. Inter-Rater Reliability

Inter-rater reliability of the INSPECT was measured for 56 first assessments and 16 second assessments separately. Results showed that the inter-rater reliability was good with an ICC of 0.78 for the first assessment and moderate with an ICC of 0.68 for the second assessment. Inter-rater reliability with ICC and 95% confidence intervals is displayed in Table 3.

#### 3.1.2. Intra-Rater Reliability

Intra-rater reliability was determined for each of the INSPECT items across all raters at time 1 and time 2. The Spearman rho correlation coefficient was moderate to excellent for 37 of 41 items and ranged from rho = 0.54 to rho = 1.0 indicating moderate to strong intra-rater reliability. The p-value for the 37 items ranged from 0.03 to <0.0001 showing statistical significance. Four items (buccal fat pads, deltoids, interosseous, and thenar muscles) had poor correlation ranging from −0.04 to 0.09 and were not statistically significant. The Wilcoxon Signed-Rank test showed no statistical differences between the first and second assessments indicating good intra-rater reliability. The intra-rater reliability results are exhibited in Table 4.

#### 3.1.3. Internal Consistency

Internal consistency for the INSPECT was found to be good to excellent with Cronbach’s α of 0.86 for the first assessment and 0.92 for the second assessment. The internal consistency results for the first and second assessments are shown in Table 2. The subset scores for the INSPECT were all ≥0.70 except for the subset of ‘preparation and initial steps’ which had a very low α of 0.18. All subset scores with alpha for each subset and the 95% confidence intervals are displayed in Table 5.

## 4. Discussion

The INSPECT is a newly designed competency tool to measure RDNs’ competence in performing NFPE on patients in an authentic workplace environment. Through a systematic, rigorous process that included both quantitative and qualitative methods with expert RDNs’ input, the INSPECT was developed and examined for reliability and validity. The preliminary validity studies utilizing the expert RDN panel had shown excellent content validation and acceptable face validation for the INSPECT [26]. This current reliability study shows equally promising results. Inter-rater reliability among 14 raters across 14 different acute care hospitals in the United States showed good reliability for first assessments and moderate level for second assessments, indicating substantial agreement among raters [30,31].

The intra-rater reliability measured with Wilcoxon Signed-Rank tests were non-significant for all 16 assessments (*p* > 0.05) demonstrating good intra-rater reliability. The intra-rater reliability correlation coefficient between time 1 and time 2 was moderate to excellent for 37 of 41 items and reached a statistical significance of *p* < 0.05. Strong correlation with a high level of statistical significance for the 37 items suggests that these items are related, and the likelihood that the items are correlating due to chance was minimal. Four items showed poor intra-rater correlation. Variability in the intra-rater reliability for these four items could have resulted from the observation of different patients at time 1 and time 2. Patients admitted to acute care have different levels of acuity ranging from acute exacerbation of chronic illnesses with the need for surgical treatment to conditions that require immediate, but relatively minor treatment. As a result, the rater may have observed the RDN perform NFPE on a patient who is cognitive and fully functional at time 1. The same rater may have observed the same RDN perform NFPE at time 2 on another patient who may not have been alert enough to follow commands to lift hands for ‘upper extremities exam’. This variation in time 1 and time 2 may mean some of the items that were indicated as ‘complete’ on a patient at time 1 may be indicated as ‘not applicable’ on a different patient at time 2 resulting in variability in assessments over time. Also, the length of stay for most patients in acute care is about 5–7 days which may mean that the same patient may not be available for observation at two weeks. Variability in intra-rater assessments may have been minimized if the same patient was examined at time 1 and time 2, however, since this study was conducted in a real-life setting, producing such a controlled setting was not feasible. A tightly controlled research environment may reduce variabilities and possibly generate the highest form of reliability, however real-life evidence from authentic clinical settings is a better representation of routine clinical practice and often produce a more meaningful clinical application [35,36,37].

The internal consistency for the INSPECT was excellent for both the first and second assessments, indicating that the items are highly correlated. A very high correlation with Cronbach’s α may also indicate redundancy of one or more items within the INSPECT [38]. Further research using exploratory factor analysis is required to determine the presence of any redundancy in the number of items. The internal consistency of 10 out of 11 INSPECT subsets showed excellent internal consistency demonstrating that the items are well correlated and are representative of the construct within each subset. One subset, the ‘preparation and initial steps’ failed to reach internal consistency. An initial impression was that the items within this subset may not belong together. Further analysis of similar items within this subset revealed that the items ‘verbal consent’ and ‘self-introduction’ correlated well, yielding a high Cronbach’s α. It may be possible to create a new subset with these two items as they appear to be related. The other three items ‘hand hygiene’, ‘utilizes personal protective equipment’ and ‘patient privacy’ did not reach sufficient α despite different combinations. Although the items within the ‘preparation and initial steps’ subset did not reach satisfactory internal consistency, these are clinically relevant items as identified by the expert RDNs in the first and second phases of the study. Hence, this subset was retained in the INSPECT without any modification. Future studies exploring the recategorization of this subset would be valuable to achieve internal consistency.

Overall, the INSPECT was found to be a reliable tool to measure RDNs’ NFPE competency. Regular competency verification of NFPE performance with the INSPECT can produce reliable results of RDNs’ competence, which in turn can aid the RDNs to accurately assess patients’ nutritional status and provide appropriate treatment and care. Competency measurement in a real-life patient care setting also will allow clinical supervisors to recognize deficiencies in NFPE learning and to plan appropriate instructional training as required. Furthermore, workplace observation presents opportunities for ongoing feedback and for customizing learning experiences at the pace of the RDN [20,39,40]. Additional studies focusing on item-level psychometrics of the INSPECT would be beneficial for continued validation in an effort to produce a robust and rigorous competency tool to measure RDN NFPE performance.

## 5. Strengths and Limitations

One of the major strengths of the INSPECT is the rigorous and comprehensive mixed-methods approach that was used to develop and validate the tool. Another major strength is that this reliability study was conducted in a multi-site setting representing an authentic clinical environment across diverse geographical locations within the United States. In addition, the INSPECT is an interactive tool providing immediate feedback in regards to the RDNs’ competence deficiencies and it can be utilized by clinical supervisors to plan individualized instructional training.

As much as it is a strength, research in real-life clinical settings can also be a limitation as it does not allow for a controlled environment. Having a controlled research setting may improve the accuracy of the INSPECT’s reliability and validity. Another limitation is that the observation of an RDN performing on the same patient at time 1 and time 2 was not feasible, which could have improved the intra-rater reliability results. Additionally, there was no training provided between time 1 and time 2 to determine if additional training would lead to improvement in assessments over time. Another limitation is that all raters who participated in this study identified themselves as of White, non-Hispanic origin, and a vast majority identified themselves as females. Although this is a potential concern, the dietetics profession in the United States is dominated by 93% female RDNs and 82% White, non-Hispanics [34]. Hence it was not surprising that the study participants were mainly made up of one gender and one ethnic group. Attempts are being made to diversify the dietetics profession to include gender diversity and various minority ethnic groups. The outlook is optimistic for future studies to include diverse participants. Additionally, future studies should be designed to offer training in areas of deficiencies identified at time 1 and competence should be remeasured at time 2 after a sufficient gap. This would help to gauge the improvement in learning over time to better understand how well the INSPECT captures the change in learning.

## 6. Conclusions

This current reliability study expands on the previous phases of the study, providing evidence for inter-rater, intra-rater, and internal consistencies of the INSPECT in authentic acute care settings. Results from the different phases of the study indicate that the INSPECT is a content validated and reliability tested tool to assess RDN’s competence in NFPE. As RDNs are increasing their scope of practice to incorporate competencies beyond their traditional scope, reliability and validity tested tools become valuable to promote and maintain RDN competence. The INSPECT is a practical tool for clinical supervisors to measure the RDNs’ mastery of NFPE competence in acute care settings, to identify deficiencies, and to offer personalized feedback and training, which in turn would ensure safe and effective nutrition assessment of patients. Future studies with a larger sample size that includes dietetic students, dietetic interns, and RDNs of different ability levels would be valuable to gain further insight into how the INSPECT distinguishes the NFPE competency levels among these groups.

## Figures and Tables

**Table 1:** Final 41 NFPE Components on the INSPECT.

INSPECT Subsets	Number of Items	NFPE Components
Preparation and Initial Steps	5	Hand Hygiene
		Personal Protective Equipment (PPE)
		Patient Privacy
		Self-Introduction
		Verbal Consent
Head and Hair Exam	2	Hair Changes
		Dry, Brittle Hair/Easily Pluckable Hair
Face Exam	3	Face & Nasolabial Areas for Flakiness
		Temporal Muscles
		Buccal Fat Pads
Eye Exam	3	Eye Conjunctivae for Pale Color
		Bitot’s Spots
		Orbital Fat Pads
Mouth and Oral Cavity Exam	5	Dentures
		Exam of Perioral Areas for Angular Stomatitis/Cheilosis
		Oral Ulcer and Lesions
		Gums and Teeth
		Tongue Inspection for Filiform Papillary Atrophy, Magenta/Beefy-Red Tongue, Glossitis
Clavicular/Thoracic Region Exam	6	Pectoralis Major
		Deltoids
		Acromion Process Protrusion
		Intercostal Muscles
		Muscles Around Midaxillary Line
		Iliac Crest Prominence and Iliac Crest Skinfolds
Back/Scapular Region Exam	2	Posterior Trapezius Muscles
		Scapular Muscles (Supraspinatus & Infraspinatus)
Upper Extremities Exam	5	Skin Exam of Upper and Lower Arm for Follicular Hyperkeratosis, Corkscrew Hair, Lanugo
		Biceps/Triceps
		Nail Exam for Color, Koilonychia, Beau’s Lines, Splinter Hemorrhage, Clubbing
		Interosseous Muscles
		Thenar Eminence
Lower Extremities Exam	4	Leg Exam for Petechiae and/or Purpura
		Quadriceps Muscles
		Gastrocnemius Muscles
		Pitting Edema
Functional Grip Strength Exam	2	Handgrip using Dynamometer When Available (Objective Measure)
		Handshake and/or Grip/Squeeze Fingers (Subjective Measure—Not Part of Academy/ASPEN Diagnostic Criteria)
Bedside Manner and Etiquette	4	Bilateral Inspection and Palpation
		Patient Dignity
		Patient Position
		Patient Interview
Total	41	

**Table 2:** Demographic Characteristics of Raters.

Variable	Median (Q1–Q3) ^	*n* = 14	%
**Age (years)**	35 (28.7–52.5)		
**Gender**			
Males		2	14
Females		12	86
**Ethnic Background**			
White, Non-Hispanic		14	100
**Highest Degree Earned**			
Bachelor’s		5	36
Master’s		9	64
**Primary Job Role**			
Regional/Clinical Nutrition Manager		11	78.6
Lead Clinical Dietitian/Specialist		3	21.4
**Primary Work Location**			
Inpatient Acute Care		14	100
**Geographic Regions**			
South		10	71.4
North East		1	7.1
Mid-West		2	14.2
West		1	7.1
**Years of Practice as Clinical Dietitian**	11.5 (5.8–17)		
**Years of Experience in Performing NFPE**	5 (3.8–5.3)		
**Years of Experience Supervising RDNs**	6 (4–10)		
**Number of RDNs Supervised**	5.5 (4–11)		

^ Q1—Lower Quartile, Q3—Upper Quartile.

**Table 3:** Inter-Rater Reliability and Overall Internal Consistency of the INSPECT.

INSPECT Assessment for 41 Items	Cronbach’s α (95% Confidence Interval)	ICC (95% Confidence Interval)
56 first assessments	0.86 (0.81–0.91)	0.78 (0.68–0.85)
16 second assessments at time 2	0.92 (0.85–0.97)	0.68 (0.41–0.86)

**Table 4:** Intra-Rater Reliability of the INSPECT.

INSPECT Items (16 Assessments at Time 1 and Time 2)	Spearman Correlation Coefficient		Wilcoxon Signed-Rank Test	
	***Rho***	***P***	***Z***	***P***
Hand Hygiene	1.0		0.000	1
Utilizes PPE	1.0		0.000	1
Patient Privacy	1.0		0.000	1
Self-Introduction	1.0		0.000	1
Verbal Consent	1.0		0.000	1
Hair Changes	0.54	0.03	−1.414	0.16
Dry, Brittle Hair	1.0	<0.0001	0.000	1
Flakiness -Nasolabial area	0.86	<0.0001	−1.000	0.32
Temporal Muscles	1.0		−1.000	0.32
Buccal Fad Pads	0.09	0.72	−1.732	0.08
Eye Color	1.0	<0.0001	0.000	1
Bitot’s Spots	1.0	<0.0001	0.000	1
Orbital Fat Pads	1.0	0.012	−1.000	0.32
Dentures	0.71	0.0014	−1.732	0.08
Exam of Perioral Areas	0.61	0.018	0.000	1
Oral Ulcer/Lesions	0.61	0.012	0.000	1
Gums/Teeth	0.61	0.012	0.000	1
Tongue Inspection	0.61	0.012	0.000	1
Pectoralis	0.68	0.003	−1.000	0.32
Deltoids	0.06	0.80	−1.414	0.16
Acromion Process Protrusion	1.0		−1.000	0.32
Intercostal Muscles	0.73	0.001	−1.000	0.32
Muscles along Midaxillary Line	1.0		−0.577	0.56
Iliac Crest Skinfolds	0.81	0.001	−1.342	0.18
Posterior Trapezius Muscles	1.0		−1.342	0.18
Scapular Muscles	0.73	0.001	−1.000	0.32
Skin Exam of Upper Extremities	1.0	0.001	0.000	1
Biceps/Triceps	1.0		−1.000	0.32
Nail Exam	0.73	0.001	−1.000	0.32
Interosseous Muscles	-0.09	0.71	−0.272	0.79
Thenar Muscles	-0.04	0.87	−0.378	0.71
Leg Inspection	0.8	0.0001	0.000	1
Quadriceps	0.63	0.008	−1.000	0.32
Gastrocnemius	0.73	0.001	−1.000	0.32
Pitting Edema	0.80	0.002	−0.447	0.66
Handgrip using Dynamometer when available (objective measure)	1.0		0.000	1
Handshake and/or Grip/Squeeze fingers (subjective measure, not part of Academy/ASPEN diagnostic criteria)	0.83	0.0001	−1.000	0.32
Bilateral Inspection & Palpation	0.61	0.01	−1.732	0.08
Patient Dignity	1.0		0.000	1
Patient Position	1.0		0.000	1
Patient Interview	1.0		0.000	1

**Table 5:** Cronbach’s Alpha of the INSPECT Subsets.

INSPECT Categories	INSPECT Items	Number of Items	Cronbach’s Alpha Subset Scores	95% Confidence Interval	
				Lower Bound	Upper Bound
Preparation and Initial Steps	Hand Hygiene	5	0.18	−0.16	0.45
	Utilizes PPE Patient Privacy				
	Introduces Self/Exam				
	Verbal Consent				
Head and Hair Exam	Hair Changes Dry, Brittle Hair	2	0.70	0.51	0.81
Face Exam	Flakiness -Nasolabial area Temporal Muscles	3	0.98	0.94	1.0
	Buccal Fad Pads				
Eye Exam	Eye Color	3	0.98	0.94	1.0
	Bitot’s Spots				
	Orbital Fat Pads				
Mouth and Oral Cavity Exam	Dentures	5	0.98	0.96	0.99
	Exam of Perioral Areas Oral Ulcer/Lesions				
	Gums/Teeth				
	Tongue Inspection				
Clavicular/Thoracic Region Exam	Pectoralis	6	0.81	0.73	0.87
	Deltoids Acromion Process Protrusion				
	Intercostal Muscles Muscles along Midaxillary Line				
	Iliac Crest Prominence and Skinfolds				
Back/Scapular Region Exam	Posterior Trapezius Muscles Scapular Muscles	2	0.81	0.70	0.88
Upper Extremities Exam	Skin Exam of Upper Extremities	5	0.98	0.95	0.99
	Biceps/Triceps				
	Nail Exam				
	Interosseous Muscles				
	Thenar Muscles				
Lower Extremities Exam	Leg Exam	4	0.97	0.92	0.99
	Quadriceps				
	Gastrocnemius				
	Pitting Edema				
Functional Grip Strength Exam	Handgrip using Dynamometer when available (objective measure)	2	0.98	0.90	1.0
	Handshake and/or Grip/Squeeze Fingers (subjective measure, not part of Academy/ASPEN diagnostic criteria)				
Bedside Manner and Etiquette	Bilateral Inspection and Palpation	4	0.92	0.77	0.99
	Patient Dignity				
	Patient Position				
	Patient Interview				

## Data Availability

The data is not publicly available to protect the confidentiality and privacy of the study participants.
